# Updating the NLRC4 Inflammasome: from Bacterial Infections to Autoimmunity and Cancer

**DOI:** 10.3389/fimmu.2021.702527

**Published:** 2021-06-30

**Authors:** Jiexia Wen, Bin Xuan, Yang Liu, Liwei Wang, Li He, Xiangcai Meng, Tao Zhou, Yimin Wang

**Affiliations:** ^1^ Department of Central Laboratory, First Hospital of Qinhuangdao, Hebei Medical University, Qinhuangdao, China; ^2^ Department of General Surgery, First Hospital of Qinhuangdao, Hebei Medical University, Qinhuangdao, China

**Keywords:** NLRC4, inflammasome, autoimmune disease, cancer, PANoptosis

## Abstract

Inflammasomes comprise a family of cytosolic multi-protein complexes that modulate the activation of cysteine-aspartate-specific protease 1 (caspase-1) and promote the maturation and secretion of interleukin (IL)-1β and IL-18, leading to an inflammatory response. Different types of inflammasomes are defined by their sensor protein which recognizes pathogenic ligands and then directs inflammasome assembly. Although the specific molecular mechanisms underlying the activation of most inflammasomes are still unclear, NLRC4 inflammasomes have emerged as multifaceted agents of the innate immune response, playing important roles in immune defense against a variety of pathogens. Other studies have also expanded the scope of NLRC4 inflammasomes to include a range of inherited human autoimmune diseases as well as proposed roles in cancer. In this review article, we provide an updated overview of NLRC4 inflammasomes, describing their composition, activation mechanisms and roles in both microbial infections and other disease conditions.

## Introduction

The innate immune system is the body’s first line of defense against invasion by pathogens and is formed during long-term evolution of the germ line. Host regulation of the innate immune response is played out by a large numbers of pattern recognition receptors (PRRs) which primarily recognize pathogens through two types of cellular interaction. One involves detection of conserved structures on pathogens, termed pathogen-associated molecular patterns (PAMPs) through membrane-bound receptors including the Toll-like receptors (TLRs) while the other involves cytoplasmic interactions between internalized pathogen-specific molecules. The latter involves retinoid acid-inducible gene I (RIG-I)-like receptors (RLRs) and nucleotide-binding oligomerization domain (NOD)-like receptors (NLRs) (1–3). Notably, there are over 20 different known NLRs in humans, acting to detect pathogens and serving as a platform for the assembly of inflammasomes.

Inflammasomes comprise high molecular weight complexes formed in the cytoplasm of cells which serve as a platform for inflammatory caspase activation. Typically, the inflammasome sensor represents a single protein species which directs inflammasome assembly, recruiting and activating caspase-1 (CASP1). Caspase-1 then cleaves and activates proinflammatory cytokines including interleukin (IL-1β and IL-18) as well as the pore-forming protein Gasdermin-D (GSDMD) (4–8), the latter inducing pyroptotic cell death to restrict pathogen replication. Inflammasomes that have been identified include NLRP1, NLRP3, NLRP6, NLRP7, NLRP12, NLRC4, and NAIP (9). Importantly from the perspective of this review which focusses on NLRC4, NAIP and NLRC4 also cooperatively form a single inflammasome as sensor (NAIP) and adaptor (NLRC4) proteins (10–14).

## Structure of NLRC4

**Figure 1:**
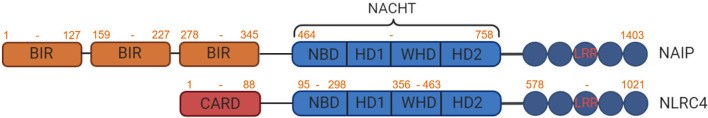
Protein domain structure of NAIP and NLRC4. NAIPs and NLRC4 share two common domains, the NACHT domain composed of nucleotide binding domain (NBD), helical domain 1 (HD1), winged helix domain (WHD) and helical domain 2 (HD2) and a C-terminal LRR. In addition, NAIP has three Baculovirus Inhibitor-of-Apoptosis repeat (BIR) domains and NLRC4 contains an N-terminal CARD.

The domain organization structures of NLRC4 and NAIP proteins are compared in **Figure 1**. Both proteins contain conserved domains present in all NLR family members which are essential for their role in inflammasomes, namely the nucleotide-binding domain (NBD) and the C-terminal leucine-rich repeat (LRR). The NBD consists of a helix domain 1 (HD1), a winged helix domain (WHD) and a helix domain 2 (HD2). This NBD-HD1-WHD-HD2 combination is often referred to as the NOD or NACHT (ADP), which occupies the NOD domain, interact with each other through a wide range of intramolecular interactions (15). Further studies have demonstrated that mutations in the key ADP proximal residue H443 leads to NLRC4 oligomerization, thereby promoting inflammasome activation. Interestingly, the majority of point mutations in NLRC4 associated with autoinflammatory syndromes (see below) are also concentrated near the ADP binding site, suggesting that nucleotide binding or ATP hydrolysis is required for oligomerization and activation of inflammasomes. Similarly, mutation of the ATP phosphate-binding loop (P-Loop) prevents NLRC4 from inducing CASP1 activation (16). The NOD domain is homologous to the AAA+ATP domain and is responsible for ATP-dependent self-oligomerization. Conversely, the LRR domain is comprised of repeated 8-15 amino acid leucine-rich helix modules, for example 15 in NLRC4, is involved in identifying ligands such as PAMP. The first 94 amino acids of NLRC4 also constitute a CARD domain typically consisting of six inversely parallel α-helices wrapped around a hydrophobic core. CARD provides a link between the splicing protein ASC and the effector molecule CASP1 which is necessary for mediating downstream signaling.

## Triggering the NLRC4 Inflammasome

**Figure 2:**
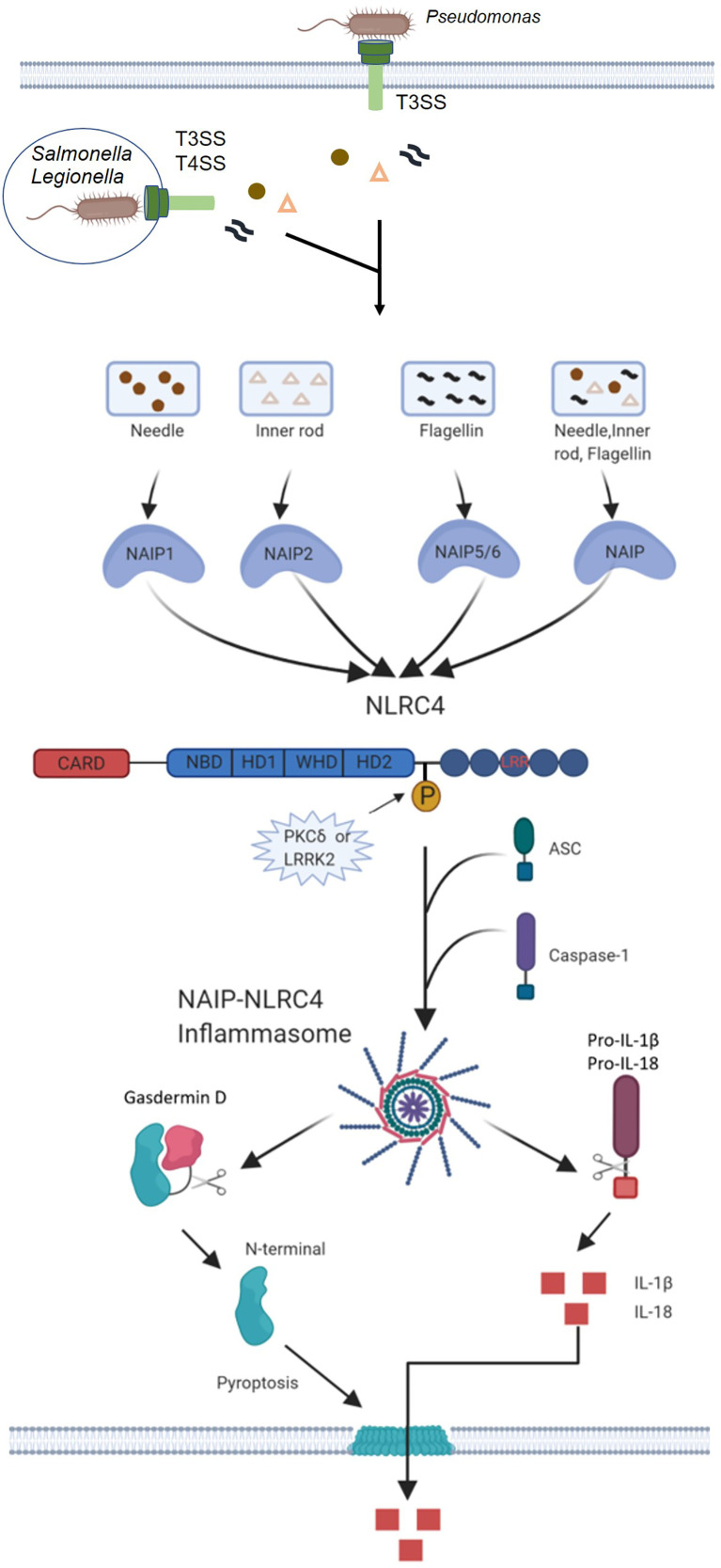
Mechanisms of NAIP-NLRC4 inflammasome activation. Infection of macrophages with various Gram-negative bacteria, including *S. typhimurium*, *L. pneumophila* and *P. aeruginosa*, activates caspase-1 *via* NLRC4. A critical step is the cytosolic delivery of flagellin or PrgJ-like proteins through bacterial T3SS or T4SS. The needle and inner rod proteins are sensed by mouse NAIP1 and NAIP2, whereas flagellin is sensed by murine NAIP5 or NAIP6. The needle and inner rod proteins and flagellin are all sensed by human NAIP. Ligand-bound NAIPs recruit NLRC4 to the same complex to drive activation of the NLRC4 inflammasome. NLRC4 acts as an adapter for caspase-1 activation. Caspase-1 cleaves the pore-forming factor GSDMD, whereby the N-terminal domain of GSDMD forms pores in the host cell membrane. Caspase-1 also cleaves the proinflammatory cytokines pro-IL-1β and pro-IL-18, generating biologically active versions of these cytokines for release through the membrane pores generated by GSDMD. The pores formed by GSDMD also lead to lytic cell death *via* pyroptosis.

Several gram-negative bacteria, including *Salmonella typhimurium*, *Legionella pneumophila*, *Pseudomonas aeruginosa* and *Shigella flexneri* induce caspase-1 activation and pyrolysis through the NLRC4 inflammasome. NLRC4 inflammasome activation requires a functional type III secretion system (T3SS) for *S. typhimurium (* 17, 18), *S. flexneri* (19) and *P. aeruginosa* (20, 21) or type IV secretion system (T4SS) for *L. pneumophila (* 22, 23). The main function of the bacterial secretion system is to form holes in the host’s membrane to mediate virulence factors (effectors proteins) into the cell cytosol (24). *S. typhimurium*, *L. pneumophila* and *P. aeruginosa* seem to induce the activation of caspase-1 through the cytoplasmic transmission of flagellin, which triggers the activation of the NLRC4 inflammasome. The inflammatory activation of caspase-1 through NLRC4 can be reproduced in the cytosol of the host by delivering purified flagellin with cationic liposomes or expression systems (17, 18, 25). Therefore, in the process of bacterial infection, the leakage of a small amount of flagellin into the host cytoplasm through T3SS or T4SS may trigger NLRC4 activation (**Figure 2**).

The general hypothesis in the field is that NLRC4 may bind certain virulence factors of flagellin and T3SS. However, no studies have been able to show direct interactions between these ligands and NLRC4. Another possibility is that NLRC4 may recruit articulator proteins for ligand recognition. Indeed, Miao and Colleagues went on to demonstrate that the component type III secretion system (T3SS) of *S. typhimurium* induces murine NLRC4-dependent CASP1 activation in the absence of flagellin, suggesting that activation by the bacterial ligand NLRC4 may be more complex (24, 26). A study has shown that NLRC4 can form heterotypic complexes with other NACHT-containing proteins (27). One NACHT-containing protein that has been shown to be associated with inflammasome activation is NAIP5, as it is required for flagellin-mediated inflammasome activation (25), but not for flagellin-independent NLRC4 inflammasome activation (28). Indeed, independent studies by Kofoed and Vance (29) and with Zhao et al. (30) have shown that NAIP proteins can act as upstream sensors for NLRC4-activating ligands (29, 30). These research teams demonstrated almost simultaneously that mouse NAIP5 and NAIP2 formed complexes with NLRC4 and flagellin or T3SS rod protein. It was later found that Naip6 could replace Naip5 in recognition of flagellin, whereas Naip1 exhibited a similar ability to induce NLRC4 inflammasome activation in response to the T3SS needle protein PrgI (31, 32). Thus, different Naip genes in mice encode proteins that confer specific ligand-mediated activation of NLRC4 in response to some bacterial ligands. Furthermore, genetic deletions of NAIP in mice also confirm their importance in mediating ligand specificity (33–35). The human genome encodes only one functional NAIP protein (hNAIP) (36). Initially, hNAIP was shown to be functionally similar to murine NAIP1, which detects T3SS needle protein (31, 32). Further studies have revealed that hNAIP can mediate NLRC4 activation in response to both T3SS components and flagellin (37, 38). In contrast to murine NAIP, only the needle protein of the Gram-negative bacterium purple bacterium Chromobacterium violaceum (CprI) has been shown to interact directly with hNAIP (30). It is unclear whether flagellin and T3SS endobar protein also directly bind to hNAIP, and how individual sensors recognize these different ligands.

Activation of the NLRC4 inflammasome is triggered by sensor activation and oligomerization, which provides a site for ASC and caspase-1 recruitment (39). The activation process can be briefly summarized as follows: interaction of inactive NAIP proteins with ligands, ligand activation of NAIP proteins, ligand-NAIP-induced interaction with inactive NLRC4 monomers, the activation of NLRC4 monomers, interaction of activated NLRC4 with inactive NLRC4 monomers, the successive addition of NLRC4 monomers (40, 41).

Structural and mechanistic studies provide in-depth understanding of NAIP-NLRC4 inflammasome assembly process. In the first step, the activating ligand interacts with the inactive NAIP protein and induces a conformational change in the NAIP protein, thereby alleviating the self-inhibitory effect. The D0 structural domain of flagellin is able to engage the six structural domains of NAIP5, forcing a change to the active conformation (42). Although other ligand-NAIPs lack structural insight, it can be assumed that they proceed through a similar mechanism of ligand-induced NAIP activation. Interestingly, analysis of mouse Naip chimeras has shown that the LRR structural domain, which is commonly thought to be important for ligand recognition, is not required for ligand binding (43). Instead, the α-helical structural domain in the NBD is required to confer ligand specificity (43).

Once activated by their respective ligands, NAIP proteins can interact with inactive NLRC4 monomers. Inactive NLRC4 monomers have been structurally characterized by X-ray crystallography and revealed that ADP-mediated interactions between the central NBD and WHD domains stabilize the closed conformation of NLRC4 (15). This study also revealed that the C-terminal LRR is located on one space of the monomeric molecule NBD and chelated NLRC4 (15). Mutagenesis that eliminates key inhibitory interactions leads to constitutive NLRC4 inflammasome activation and bypasses the need for flagellin (15). Once the ligand-bound NAIP complex interacts with NLRC4, it is sufficient to trigger a conformational change in NLRC4 that pushes it into the active conformation (40, 41). The activation of the NLRC4 monomer is achieved by rotating the conformation of the hinge region between HD1 and WHD by 90°. This conformational change exposes the basic “catalytic surface” on the active NLRC4, which can interact with the acidic “acceptor surface” on the incoming inactive NLRC4 monomer. This interaction activates a second NLRC4 monomer, which can recruit more NLRC4 monomers and lead to a self-propagating mechanism that results in the formation of a 10-12 subunit whorl (40, 41). Importantly, NAIPs also possess a “catalytic surface” that matches the “receptor surface” of NLRC4 and enables initiation of oligomerization, but they do not possess a “receptor surface” and therefore only a single NAIP is found per NAIP-NLRC4 inflammasome complex (40, 41). This difference indicates that NAIP-NLRC4 inflammasome can respond to lower concentrations of activating ligands, and different cell death pathways have different thresholds.

## Regulation of NLRC4

NLRC4 activation is also influenced by regulatory mechanisms, mainly including transcriptional regulation and posttranslational modifications, particularly phosphorylation and possibly ubiquitination (44, 45). A study showed that both NAIP and NLRC4 are transcriptionally induced by interferon regulatory factor (IRF)8 (46). IRF8 acts as a transcriptional activator of murine NAIP 1, 2, 5, and 6 and NLRC4. It was also found that NLRC4 inflammasome activation in Irf8−/− BMDMs was not completely abrogated, suggesting that additional factors contribute to the transcriptional regulation of NAIPs and NLRC4 (46). In addition, no IRF8 peak was found in chromatin immunoprecipitation sequencing data at the NAIP1 locus, but IRF8 peaks were found at the NAIP2, NAIP5, and NAIP6 loci (46). It is possible that NAIP1 may be regulated differently to the other NAIPs.

To investigate the phosphorylation modifications of NLRC4 during activation, Qu et al. (47)conducted experiments in genetically modified mice expressing NLRC4 with a carboxy-terminal 3×Flag tag. Infection with *S. typhimurium* induced phosphorylation in NLRC4 at serine 533 which could not be prevented by CASP1 inhibition, clearly suggesting that this phosphorylation event occurred upstream of CASP1 cleavage. In addition, protein kinase Cδ (PKCδ) and leucine-rich repeat kinase 2 (LRRK2) were found to phosphorylate NLRC4 (48). However, it has been reported that flagellin can induce NLRC4 phosphorylation, but not NLRC4 inflammasome activation (49), raising questions as to the whether these NLRC4 kinases play a dominant role in NLRC4 activation.

## NLRC4 Effector Mechanisms

Initially, it was thought that NLRC4 induced inflammation through the activation of CASP1, thereby promoting the conversion of pro-IL-1β and pro-IL-18 to active IL-1β and IL-18. CASP1 also activates a cytoplasmic protein known as GSDMD, whose cleaved N-terminal domain acts on the host cell membrane, resulting in loss of membrane integrity and ultimately inducing cell scorching (pyroptosis) (50). Unlike apoptosis, pyroptosis occurs more rapidly, and inflammatory factors such as IL-1β and IL-18 can be released to the outside of the cell through the membrane pore formed by GSDMD to stimulate the body’s defense against pathogens (**Figure 2**). In addition, Moltke et al. (51) found that an artificial flagellin-derived NAIP5-NLRC4 activator releases arachidonic acid through the activation of calcium-dependent phospholipase A2 (PLA2). Arachidonic acid activated by CASP1 promotes the rapid synthesis of prostaglandins and leukotrienes. The mechanism of arachidonic acid release and whether this mechanism is related to the activation of NLRC4 is unclear. Proteomic research has identified as many as 1,000 potential targets of CASP1, indicating the strong likelihood that CASP1 activates many other downstream signals in association with NLRC4 inflammasome activation in addition to the above-mentioned signal transduction pathways.

The CARD domain of NLRC4 directly interacts with pro-CASP1 to catalyze CASP1 activation, and the apoptosis-associated speck-like protein (ASC) plays a positive role in the activation process. Other proteins inflammasome constituents including NLRP3, PYRIN, and AIM2 also interact with ASC to recruit and induce cleavage of pro-CASP1 into active P10/P20 tetramers, resulting in the cleavage of pro-IL-1β and pro-IL-18 into their active forms and release into the extracellular to participate in immune response (52). The biological activities of IL-1β and IL-18 and pyroptosis are mainly beneficial to the host during the infection process. However, IL-1β and IL-18 induced by endogenous danger signals can trigger aseptic inflammation, which is a risk factor for autoinflammation and metabolic diseases. Therefore, the activation of the inflammasome must be carefully controlled to avoid overt tissue damage. Here, we outline the latest developments in the field of inflammasome biology, with particular emphasis on the regulation of inflammasome signals in health and disease (53, 54).

## NLRC4 Inflammasome and Bacterial Infection

Some bacterial infections activate NLRC4 inflammasomes causing an inflammatory response. Moderate inflammation facilitates the clearance of bacteria and inhibits bacterial infection, while excessive inflammation causes host cell death. Certain bacteria can survive intracellularly and can dampen the activation of NLRC4 inflammasomes, thus evading detection by the host immune system and causing severe infections.

### NLRC4 Inflammasome and *S. typhimurium*

The NLRC4 inflammasome has been reported to be important in controlling *S. typhimurium* infection, the causative agent of salmonellosis. Mariathasan et al. (55) found that NLRC4-deficient murine macrophages fail to activate CASP1 after exposure to *S. typhimurium*, consistent with the findings of Franchi et al. who found that caspase-1 activation was NLRC4-dependent (17, 18). Activated caspase-1 shears pro-IL-1β and pro-IL-18 to induce pyroptosis and caspase-7 activation, leading to an inflammatory response (56, 57). However, other studies observed that infecting NLRC4-overexpressing human cell lines with a moderate load of *S. typhimurium* inhibited the proliferation of *S. typhimurium*. On the other hand, macrophages overexpressing NLRC4 die when they were infected with a high load of *S. typhimurium* (58). These findings suggest that NLRC4 inflammasomes may play different roles depending on the bacterial load.

Most studies usually employ macrophages to investigate the role of inflammasomes in innate immune responses. Instructively unlike macrophages, *S. typhimurium* infection does not induce pyroptosis in neutrophils although there is activation of NLRC4 inflammasomes. The discovery of this anti-pyroptotic mechanism in neutrophils is considered a new inflammasome mechanism (59). Nonetheless, even though neutrophils do not succumb to rapid cell death by pyroptosis, they do continue to release pro-inflammatory IL-1β at the site of infection to promote inflammation and direct the immune response. Therefore, this finding also shows that the NLRC4 inflammasome pathway in neutrophils can maximize the host’s pro-inflammatory response and antibacterial effects (60–64). Recent studies have also found that the activation of NLRP3 is related to NLRC4 in macrophages infected with *S. typhimurium* or transfected with *S. typhimurium* flagellin. This interaction between NLRP3 and NLRC4 reveals possible functional overlaps between different inflammasomes (65).

### NLRC4 Inflammasomes and *S. flexneri*

*S. flexneri* is the causative agent of bacterial dysentery. After macrophage infection, caspase-1 is activated with subsequent cleavage of IL-1β and resulting pyroptosis. This process is not dependent on flagellin but requires the involvement of T3SS and NLRC4 inflammasomes (19, 66). Suzuki et al. found *S. flexneri* induces rapid macrophage cell death by delivering the invasion plasmid antigen H7.8 (IpaH7.8) enzyme 3 (E3) ubiquitin ligase effector *via* the type III secretion system, thereby activating the NLRP3, NLRC4 inflammasomes and caspase-1 and leading to macrophage cell death in an IpaH7.8 E3 ligase-dependent manner (67). These findings indicate that *S. flexneri* can activate NLRC4 inflammasomes through multiple T3SS mechanisms. However, *S. flexneri* can also evade the innate immune system through inhibiting the activation of NLRC4 inflammasomes. Indeed, Hermansson et al. demonstrated that *S. flexneri* can regulate inflammasome activation and the inflammatory response after infection of different cells such as epithelial cells, macrophages, neutrophils, dendritic cells and lymphocytes. They suggest that *S. flexneri* optimizes it interaction with the host to successfully establish infections by regulating inflammasome activation (68).

### NLRC4 Inflammasomes and *L. pneumophila*

*L. pneumophila* is a flagellated, chimeric intracellular bacterium causing Legionnaires’ disease (69–71). It has been shown that NAIP5 and NLRC4 act synergistically to induce caspase-1 activation in a *L. pneumophila* mouse model (28), and that NAIP5/NLRC4 inflammasomes are activated by *L. pneumophila* flagellin, which subsequently causes caspase-1 activation and pyroptosis. *L. pneumophila* infection can cause activation of both caspase-1 and caspase-7 (72) although the mechanism of action between NAIP5 and NLRC4 inflammasomes is not well defined. The two main possibilities involve formed NAIP5 inflammasomes interacting with NLRC4 inflammasomes, or by alternatively that NAIP5 acting as a cytoplasmic receptor to promotes the activation of NLRC4 inflammasomes. Moreover, at low *L. pneumophila* (physiological) infection loads in epithelial cells it has been reported that caspase-1 activation is dependent on bacterial flagellin and host NLRC4 whereas at high bacterial loads caspase-1 activation is independent of NLRC4 (73). This also indicates that the role of NLRC4 inflammasomes in caspase-1 activation varies among *L. pneumophila* infection vectors. Recent studies have shown that, in addition to the well-studied NLRC4-NAIP5 flagellin recognition pathway, tumor necrosis factor (TNF) and reactive oxygen species (ROS) play important roles in the effective control of intrinsic immunity to *L. pneumophila* infection, functioning independently of NLRC4, caspase-1, caspase-11 and NOX2. Thus a number of signaling pathways, including NLRC4 inflammasome, are activated and interact with each other after *L. pneumophila* infection *in vivo* (74).

### NLRC4 Inflammasomes and *P. aeruginosa*

*P. aeruginosa* causes severe infections in hospitalized patients with acute disease. It activates NLRC4 inflammasomes through T3SS and induces host tissues to produce inflammatory cytokines such as IL-1β and IL-18. Among them, *P. aeruginosa* virulence factor ExoU is the effector protein of T3SS (21). It has also been reported that flagellin is involved in the activation of caspase-1 after *P. aeruginosa* infection under low bacterial load (21). However, unlike *L. pneumophila*, *P. aeruginosa* mutants lacking flagellin still depend on NLRC4 for activation of caspase-1. This indicates that NLRC4 recognizes PAMPs other than flagellin, but the specific mechanisms need further study.

### NLRC4 and *L. monocytogenes*

Listeria monocytogenes is a flagellated Gram-positive bacterium that causes meningitis and sepsis. *L. monocytogenes* engulfed by macrophages can escape from phagosomes and replicate in their cytoplasm. The presence of *L. monocytogenes* in the cytoplasm activates caspase-1, processes and secretes IL-1β and IL-18, and causes caspase-1-dependent pyroptosis (75).

## Panoptosis in Microbial Infections

The innate immune system recognizes microbial molecules that are conserved in many pathogens and responds rapidly by producing inflammatory mediators and activating programmed cell death pathways with resulting responses including pyroptosis, apoptosis and necroptosis. The binding of pattern recognition receptors to inflammatory cytokines is activated by receptor-induced signaling containing the death domain, triggering a highly relevant cell death process called PANoptosis (76). PANoptosis is a unique, physiologically relevant, inflammatory programmed cell death pathway regulated through the PANoptosome which effector molecules from the pyroptotic, apoptotic, and necroptotic cell death pathways interact to form a single molecular complex (77). Depending on the proximal sensors of different microbial infections, one or more sensor molecules initiate PANoptosome formation, which then serves as molecular scaffold for the interaction and activation of inflammasome in septic diseases (e.g., as inflammatory vesicle sensors, ASC and caspase-1), apoptosis (caspase-8) and necroptosis (RIPK3, RIPK1) (77–81). Immunoprecipitation confirmed that caspase-8, ASC, RIPK1 and ZBP1 can interact with NLRP3 suggesting the formation complexes between the PANoptosis molecules (80).

To date, although PANoptosis has most often been described in conjunction with NLRP3 inflammatory vesicle activation (82–85), multiple lines of evidence suggest that NLRC4 may also play a key role in PANoptosis. Caspase-mediated cleavage of the DNA damage sensor poly (ADP-ribose) polymerase 1 (PARP1) is a hallmark of apoptosis. Activation of NLRP3 and NLRC4 inflammasome both induces processing of full-length PARP1 into an 89 kDa fragment. Macrophages lacking Casp1, Nlrp3, Nlrc4 or Pycard are also unable to cleave PARP1, suggesting that protease-mediated inactivation of PARP1 is a shared feature of apoptotic, necrotic, and pyroptotic cells (86); however, the proposed role of NLRC4 in PANoptosis requires further substantiation.

Recently, it was shown that *S. typhimurium* activates and regulates PANoptosis (77). Typical pyrolytic ligands (including T3SS needle protein and flagellin) both activate NAIP-NLRC4 inflammasome (18, 87, 88). *S. typhimurium* T3SS effector proteins (AvrA, SspH1, SseL, GtgA, SvC, SopB, SseK1/4) broadly inhibit TLR-mediated and TNF-mediated inflammatory signaling. *S. typhimurium* effector proteins may balance the production of inflammatory mediators and therefore may trigger PANoptotic cell death responses in some cells (89). Despite the secretion of many immunomodulatory effectors by *S. typhimurium*, NLRC4 inflammatory vesicle activation still readily occurs and can recruit NLRP3 into the combined inflammatory vesicle complex (60), which could potentially recruit other PANoptosome components to drive cell death. A recent study also identified the activation of caspase-1 and GSDMD (pyroptosis), caspase-8, caspase-7 and caspase-3 (apoptosis) and mixed-spectrum kinase structural domain-like pseudokinase (MLKL) (necroptosis) during *S. typhimurium* infection (77). Combined deletion of the PANoptotic molecules Casp1, Casp11, Ripk3 and Casp8 protects macrophages from cell death during *S. typhimurium* infection. However, deletion of individual cell death components did not completely prevent cell death, suggesting a crucial role for PANoptosis during *S. typhimurium* infection (77). Further experimental evidence is needed to elucidate the link between NAIP/NLRC4 and the molecules of the PANoptosome complex and to fully understand the role of NLRC4 in PANoptosis for host defense mechanisms.

## Bacterial Evasion Mechanisms

Pathogens have been interacting with their hosts throughout evolution, driving the development of multiple defense strategies against invading bacteria, parasites, and viruses. To counter, pathogens have also developed mechanisms to avoid recognition by the innate immune system, including activation of inflammasomes. Indeed, effective activation of inflammasomes in vertebrates is essential for controlling bacterial replication and protecting the host.

Various bacterial species including *S. typhimurium*, *Yersinia*, *L. monocytogenes* and *P. aeruginosa*, have developed strategies to inhibit inflammasome activation, including NLRC4 inflammasomes. *S. typhimurium* expresses two T3SS encoded by pathogenic islands SPI-1 and SPI-2, both of which are required for pathogenicity. SPI-1 is required for invasion of intestinal epithelial cells, whereas SPI-2 is required for replication in tissue macrophages (88, 90). T3SS expressed by SPI-1 is responsible for the injection of flagellin and PrgJ into the cytoplasm and activation of the NLRC4 inflammasome (18, 26). On the other hand, to promote bacterial replication in macrophages, *S. typhimurium* expresses the T3SS encoded by SPI-2, whose rod protein SsaI is not recognized by the NLRC4 inflammasome. Binding is prevented by a few amino acid modifications in the Ssa1 protein region recognized by NAIP2, which inhibits NLRC4 activation. In addition, *S. typhimurium* also evades NLRC4 recognition by inhibiting flagellin expression in the intracellular environment when SPI-2 T3SS is activated (26).

*P. aeruginosa* expresses T3SS in order to inject effector molecules into the host cell. These bacteria express four effector molecules, ExoU, ExoY, ExoT and ExoS, involved in triggering inflammatory responses that may lead to cell death (91). *P. aeruginosa* induces caspase-1 activation in NLRC4-dependent way, but independently of flagellin. Instructively, NLRC4-deficient macrophages are resistant to cell death after *P. aeruginosa* infection and display impaired IL-1*β* secretion (90, 91). *P. aeruginosa* lacking the effector ExoU induced NLRC4-dependent macrophage death. On the other hand, bacteria expressing ExoU killed macrophages independently of caspase-1, suggesting that ExoU blocks activation of the inflammasome (21). In vitro studies and *in vivo* lung infections indicate that caspase-1 activation and cytokine secretion depend on effective T3SS and moreover that ExoU phospholipase A2 activity is required to inhibit caspase-1 activation, but the mechanism needs further study (21, 92).

*In vitro* studies have indicated that *L. monocytogenes* infections activate NLRC4 but only poor activates NLRC4 *in vivo*. In unprovoked macrophages, NLRC4 has been shown to be important in controlling infection. Following LPS stimulation, caspase-1 activation and cell death do not require NLRC4, whereas NLRP3 plays a major role (93). *L. monocytogenes* is able to evade the immune system through different mechanisms. For example, bacteria avoid NLRC4 activation by suppressing flagellin expression through the transcriptional regulator MogR (94, 95). In contrast to other Gram-negative bacteria (e.g., *S. typhimurium*), *Yersinia* does not strongly activate NLRC4 inflammasomes (96, 97), YopK, an effector protein secreted by *Yersinia* pestis into the host cytoplasm *via* T3SS, has been shown to bind to T3SS and block NLRC4 activation. It is unclear whether YopK prevents the secretion of bacterial components into the host cytoplasm or binds to transposons into the cytoplasm and blocks NAIP recognition, or may even have other functions. In *Yersinia* pseudotuberculosis strains lacking YopK, cystatinase 1 is activated in an NLRP3/NLRC4/ASC-dependent manner and the bacteria are more susceptible to host responses *in vivo* (96). Thus, the mechanisms whereby pathogens evade immune recognition are strongly dependent upon the species and indeed the strain of microorganism involved. As highlighted by the above examples, researchers also need to be aware of the discrepancies in experimental systems, particularly the *in vitro* and *in vivo* differences which have been reported. Nonetheless, a better understanding of these evasion mechanisms is critical in order to develop improved treatment strategies.

## NLRC4 Inflammasomes and Human Auto-Inflammatory Diseases

NLRC4 has also been found to play an important role in driving human autoinflammatory disease, those diseases caused by systemic or organ-specific inflammation that is not attributable to infection, malignancy, or antigen-specific autoimmunity (98). This disease state was originally defined as a monogenic IL-1β-blocking disease associated with spontaneous or enhanced inflammasome activation. The expansion of research in this area has uncovered PYRIN and NLRP3 inflammasomes and related autoinflammatory diseases such as familial Mediterranean fever (FMF) and hypothermic inflammation-related periodic fever syndrome (CACFS). Cryopyrin-associated periodic syndromes (CAPS) and NLRC4-related autoinflammatory diseases were first described in 2014 (see below).

Several studies have now identified that mutations in the NLRC4 gene promote a gain of function resulting in the spontaneous inflammasome activation, this causing pediatric enteritis and recurrent macrophage activation syndrome (MAS) (99–101). The latter syndrome is characterized by persistently elevated serum ferritin and blood coagulation disorder (102), which is clinically similar to fatal hemophilic lymph histiocytosis. This represents a serious complication of chronic rheumatism in children and is often classified as an immunodeficiency. MAS often causes rheumatism, especially systemic juvenile idiopathic arthritis (JIA) and adult Still’s disease (adult onset still’s disease, AOSD) which is more common than systemic JIA. Although IL-1β and IL-18 are expressed and released by bone marrow cells when inflammasomes are activated, significant amounts of IL-1β and IL-18 were not detected in the patients sera with infectious diseases prior to the discovery of the NLRC4-MAS connection (103, 104). Therefore, although NLRC4 is associated with infectious diseases and MAS, high levels of IL-18 seem to be only associated with the latter, and patients with refractory severe MAS have better therapeutic effects after using IL-18 blockers and gamma interferon blockers (a cytokine induced by IL-18) (105).

Unlike other inflammatory bowel diseases, NLRC4-related enterocolitis cases occur in early infancy. Inflammation can affect the entire intestine from the stomach to the colon although patients with duodenal disease have milder symptoms (100, 106). Common NLRC4 mutations involve V341A, T337S, T337N and S171F (100, 107, 108). with notably amino acid positions 337 and 341 located in the NOD domain which is crucial for inflammasome formation (100, 109). X-ray crystallography analysis of NLRC4 shows that amino acid position 341 plays an important role in the conversion of ADP to ATP, with its mutation affecting the inflammasome activation process (100). Amino acid position 337 can interact with amino acid positions 170 and 173 (106) during inflammatory activation and stabilize the ADP binding site (107). It is speculated that the substitution of phenylalanine for serine at position 171 alters this interaction, thereby phenocopying the effects of the 337 mutation, leading to inflammasome activation and the manifestation of NLRC4-related enterocolitis.

Strikingly, although infants with colitis maintain high levels of MAS and serum IL-18, gastrointestinal diseases in patients who survive infancy can be cured (100, 101). Therefore, early intestinal colonization may promote the production of cytokines in patients with NLRC4-related enterocolitis, and the natural outcome of the disease may be related to intestinal maturation, intestinal mucosal immunity and intestinal flora. The role of NLRC4 in intestinal defense shows that there are complex interactions between NLRC4 and IL-18, intestinal microflora and immune balance.

In addition to the connection with NLRC4, MAS and enterocolitis, another study also reported that a H443P NLRC4 mutant is present in children with FACS4 (110), an autoinflammatory disease. Another familial autoinflammatory phenotype related to a missense mutation in NLRC4 resulted in symptoms including urticaria and nodular rash, conjunctivitis and arthritis (111). Other disease causing NLRC4 missense mutations have also been reported. Kawasaki et al. (112) recorded a case of a patient with CAPS, whose constitutional mosaicism had a novel NLRC4 mutation. In these patients, the specificity of NLRC4 activation for arachnoid formation has not been evaluated, but inhibition of cyclooxygenase has been shown to be ineffective except for patients with the mildest pathologies. Other studies have also shown there is a correlation between the amino acid position of the NLRC4 mutation and the autoinflammatory phenotype. Unal et al. (113) found a W374X mutation in a child with JIA and recurrent MAS. In addition, mutations at positions 171 and 177 of the nucleotide binding domain (NBD) drive CASP 8-dependent apoptosis in a human lung epithelial cell line (45). Nucleotide binding domain (NBD) mutations at positions 171 and 177 also lead to uterine recurrent macrophage activation syndrome, placental thrombosis and CAPS.

Until now, all known disease associated NLRC4 mutations affect the ADP/ATP binding domain. Moreover, research involving NLRC4 immune regulation has mainly focused on using gene knockout models in mice to investigate the role of NLRC4 in activating CASP1 in various bacterial infections. Consequently, the mechanisms of NLRC4 and related signaling pathways in common diseases such as JIA and AOSD and host defense need further research and verification.

## NLRC4 in Cancer

The differential but variable expression of NLRC4 has been observed in a variety of tumor tissues. For example, NLRC4 mRNA levels is reduced in colorectal cancer compared with normal adjacent tissues (114) but is increased in breast cancer, stomach cancer and glioma (115–117). However, other recent studies have shown that NLRC4 mRNA levels are unchanged in lung tumors (118) and hepatocellular carcinomas (119). Hence neither the reported expression or indeed role of NLRC4 in tumor regulation and suppression is always consistent, even in the same tumor type (**Table 1**).

**Table 1:** The role of NLRC4 in cancer using mouse models.

Cancer model	Mouse	Phenotype	Reference
**AOM/DSS Model of Colitis-Associated Cancer**	Nlrc4^-/-^	Increased tumor formation Reduced apoptosis within tumors Increased proliferation of colonic epithelial cells	(120)
	Nlrc4^-/-^	No increase in hyperplasia or tumor numbers	(121)
	Naip1^-6Δ/Δ^	Increased tumors within colon compared with non-littermate or littermate Naip1^-6fl/fl^ mice Impaired ability to attenuate STAT3 hyperactivation	(35)
	Naip1^-6Δ/ΔLysm^	No difference in tumor burden compared with littermate Naip1-6^fl/fl^ mice	(35)
**AOM Model of Colitis-Associated Cancer**	Naip1^-6Δ/Δ^	Increased number of tumors compared with non-littermate Naip1-6fl/fl mice	(35)
**Melanoma**	Nlrc4^−/−^	Enhanced tumor growth Reduced production of cytokines and chemokines	(122)
	Nlrc4^−/−^	No difference in tumor incidence when compared with littermate WT mice	(123)
**Mammary**	Nlrc4^−/−^	Normal diet: • Decreased tumor size compared with non-littermate WT mice High-fat diet: •Decreased tumor size •Reduced CD45+ tumor-infiltrating leukocytes •Reduced angiogenesis and VEGF-A production	(124)

Many of the studies related to NLRC4 in cancer have employed knockout mouse models. In a chemically induced colon carcinoma model employing the DNA damaging agent azomethane and dextran sodium sulfate, it was found that compared with WT mice, Nlrc4-/- mice exhibited increased tumor formation, decreased tumor apoptosis, and increased colonic epithelial cell proliferation in the early stages of the disease. Similar sensitivity to tumorigenesis was also observed in Casp1/11-/- mice, suggesting that NLRC4 inflammasomes mediate protection in this disease context (120). Other studies found enhanced tumor growth in Nlrc4-/- mice after subcutaneous injection of mouse B16F10 melanoma cells, suggesting that NLRC4 is important for regulating signaling pathways responsible for tumor growth control (122). In addition to this study, it was found that the production of cytokines (IFN-γ) and chemokines (CXCL9, CXCL10, CXCL16, and CCL5), which contribute to tumor growth, were reduced in Nlrc4-/- mice. In contrast, a recent study found no difference in tumorigenesis between littermate WT and Nlrc4-/- mice (123). This phenomenon may occur due to different genetic backgrounds, such as the generation of Nlrc4-/- mice on different sublines of C57BL/6, resulting in differences in tumor suppression (122, 125).

Another aspect linking NLRC4 and IL-1β with cancer involves their role in promoting the progression of diet-induced mammary tumors in obese mice (124). The obese tumor microenvironment favors the recruitment of macrophage and the activation of NLRC4 inflammasomes in these cells promotes the production of adipocyte-originated growth factor vascular endothelial growth factor-A (VEGF-A), thereby promoting angiogenesis (124). Similarly in fatty liver disease-induced metastasis, cell expansion and VEGF-A production are also mediated by NLRC4, which is also driven by the production of IL-1β (126). Indeed, studies of both human breast cancer patients and glioma patients have shown a similar relationship between higher NLRC4 expression and poor prognosis (35, 121). These studies also found that increased IL-1β expression may drive tumor progression. Therefore, further studies are needed to explore the preliminary link between NLRC4-mediated IL-1β secretion and tumor progression, potentially positioning the NLRC4-IL-1β pathway as a potential therapeutic target for inhibition of glioma and other cancers.

## Conclusions and Perspectives

Research over the past ten years has greatly improved the understanding of the function of the NLRC4 inflammasome, showing their function is not limited to immune recognition but rather their wider involvement in the regulation of immune responses. Indeed, the NLRC4 inflammasome is critical for fighting microbial infection and maintaining homeostasis but importantly this role can vary according to the pathogen type and infection load. Another key concept that has emerged is the discovery that not only a single NLRC4 inflammasome plays an immunomodulatory role in bacterial infections, but that interactions between inflammasomes are also an important for immune regulation. Finally, the recognition that NLRC4 dysfunction underlies many human diseases such as MAS, infantile small bowel colitis, tumors and diabetic nephropathy, has important clinical implications, providing new opportunities to both treat and even prevent these diseases. However, despite this progress, the specific activators and signaling pathways of NLRC4, the role of NLRC4 in different immune cells and immune organs, and the treatment of human NLRC4-related diseases still need to be further explored.

## Author Contributions 

YW designed the review article. J.W. wrote the manuscript and prepared the figures and tables. BX, YL, LW, LH, XM, and TZ revised the manuscript. All authors contributed to the article and approved the submitted version.

## Funding

This work was supported by the National Natural Science Foundation of China (Grant No. 81802372), the Natural Science Foundation of Hebei Province (Grant No. H2020107005 & H2020107002) and the Scientific and Technological Project of the Hebei Province of China (Grant No. 14397702D).

## Conflict of Interest

The authors declare that the research was conducted in the absence of any commercial or financial relationships that could be construed as a potential conflict of interest.
